# Dataset on comparing the corrosion indices of alum and ferric chloride coagulants with phosphate dose elevation

**DOI:** 10.1016/j.dib.2018.08.007

**Published:** 2018-08-09

**Authors:** Mohammad Hadi Dehghani, Reza Ghanbari, Zoha Heidarinejad

**Affiliations:** a Department of Environmental Health Engineering, School of Public Health, Tehran University of Medical Sciences, Tehran, Iran; b Institute for Environmental research, Center for Solid Waste Research, Tehran University of Medical Sciences, Tehran, Iran; c Department of Environmental Health Engineering, Faculty of Health, Hormozgan University of Medical Sciences, Bandar Abbas, Iran

**Keywords:** Corrosion indices, Alum and ferric chloride coagulants, Water

## Abstract

The aim of this data was to assess and compare the corrosion indices of alum and ferric chloride coagulants in conventional coagulation process of water with elevation of phosphate dose. After preparing synthetic water samples, jar experiments were performed with elevation of different phosphate doses using alum and ferric chloride coagulants. Then, corrosion indices of Ryznar and Langelier of water samples were calculated. The results indicated that the values of Ryznar and Langelier index in the experimental samples were 7 and less than zero, and the water conditions were under saturated. The corrosion and precipitation indices indicated that the water samples can be considered as corrosive waters.

**Specifications Table**

**Table:** 

Subject area	Water chemistry
More specific subject area	Corrosion and coagulation
Type of data	Tables, Figures
How data was acquired	After preparing the synthetic water samples, different doses of phosphate were injected into jar glasses, 10 mg/L alum and ferric chloride were also added to the samples and finally the phosphate level of the samples was measured by DR/2000 Direct Read Spectrophotometer at the wavelength of 420 nm. Ryznar and Langelier corrosion indices were calculated for samples.
Data format	Raw, analyzed
Experimental factors	The qualitative parameters of the samples including alkalinity, electric conductivity, total suspended solids, temperature, bicarbonate and pH were performed by the instructions in Standard methods for the examination of water and wastewater, 20th edition [1], [2], [3], [4].
Experimental features	The levels of physico-chemical parameters of the samples were determined.
Data source location	Tehran University of Medical Sciences, Tehran, Iran.
Data accessibility	The data are available with this article

**Value of the data**

• The data can be beneficial for improving the quality of potable water with corrosion problems resulting from alum and ferric chloride coagulants.
• The data can be helpful in the design and operation of conventional and advanced coagulation units in water treatment plants.
• The data comparing the Langelier and Ryznar corrosion indices of alum and ferric chloride with elevation of phosphate dose in water samples indicated that the water conditions in the samples were under saturated.

## 1 Data

The datasets include 4 Tables and 8 Figures. The water corrosion indices used are summarized in Table 1. Table 2 indicates the mixing conditions and retention time of stages of coagulation, flocculation, and sedimentation in jar experiments. The results of jar test for assessing the effect of orthophosphate dose in alteration of water corrosion indices for alum and ferric chloride coagulants are presented in Table 3, Table 4.

**Table 1:** A summary of the corrosion and water sedimentation indices used in this study [5], [6], [7], [8], [9], [10], [11], [12].

	Equation			Index value	Water condition			
Langelier saturation	LSI = pH – pHs			LSI > 0	Super saturated, tend to precipitate CaCO_3_			
index (LSI)	pHs = A + B – log (Ca2+) – log			LSI = 0	Saturated, CaCO_3_ is in equilibrium			
	(Alk) pH <= 9.3							
	pHs = (9.3 + A + B) – (C + D)			LSI < 0	Under saturated, tend to dissolve solid CaCO_3_			
	(3) pH > 9.3							
Ryznar stability	RSI = 2pHs – pH			RSI < 6	Super saturated, tend to precipitate CaCO_3_			
index (RSI)				6 < RSI < 7	Saturated, CaCO_3_ is in equilibrium			
				RSI > 7	Under saturated, tend to dissolve solid CaCO_3_			

A = (Log10 (TDS) - 1) / 10 B = -13.12 × Log10 (°C + 273) + 34.5

C = Log10 (Ca^2^ as CaCO3) - 0.4 D = Log10 (Alkalinity as CaCO3)

**Table 2:** The conditions of mixing and retention time of coagulation, flocculation, and sedimentation stages.

Parameter	Flash mixing	Flocculation	Sedimentation
Mixing speed (rpm)	120	20	–
Time (minute)	2	20	30

**Table 3:** The results of jar test for evaluating the effect of orthophosphate dose in altering the water corrosion indices; type of coagulant: aluminum sulfate (alum).

**Sample**	**Ryznar stability Index (RSI)**	**Langelier saturation index (LSI)**	**pHs**	**pH**	**TDS (mg/L)**	**EC (μmhos/cm)**	**T (**^**0**^**C)**	**HCO3**^-^ (mg/L)	**ALK mg/L (CaCo**^**3**^**)**	**Phosphate Dose mg/L (Po**^**4**^**)**
Raw water	8.22	−0.31	7.91	7.6	276.48	432	18.5	115.9	95	–
Jar 1	8.67	−0.74	7.94	7.2	279.68	437	19.1	109.8	90	0
Jar 2	8.65	−0.73	7.93	7.2	279.04	436	19.1	109.8	90	0.5
Jar 3	8.73	−0.79	7.94	7.15	279.68	437	19.2	106.75	87.5	1
Jar 4	8.78	−0.84	7.94	7.1	279.04	436	19.2	106.75	87.5	1.5
Jar 5	8.75	–0.83	7.93	7.1	279.68	437	19.1	109.8	90	2.5
Jar 6	8.76	–0.83	7.93	7.1	279.68	437	19.2	108.58	89	3.5

**Table 4:** The results of jar test for evaluating the effect of orthophosphate dose in changes of water corrosion indices; type of coagulant: ferric chloride.

**Sample**	**Ryznar stability index (RSI)**	**Langelier saturation index (LSI)**	**pHs**	**pH**	**TDS (mg/L)**	**EC** (**μmhos/cm)**	**T (**^**0**^**C)**	**HCO**_**3**_^-^**(mg/L)**	**ALK mg/L (CaCo**_**3**_)	**Phosphate Dose (mg/L Po**_**4**_**)**
Raw water	8.33	−0.37	7.97	7.6	277.12	433	17	115.9	95	–
Jar 1	8.84	−0.87	7.97	7.1	279.68	437	17.1	106.75	87.5	0
Jar 2	8.85	−0.88	7.98	7.1	280.68	438	17	106.75	87.5	0.5
Jar 3	8.88	−0.89	7.99	7.1	280.32	437	17.1	103.7	85	1
Jar 4	8.86	−0.88	7.98	7.1	279.68	437	17.1	106.75	87.5	1.5
Jar 5	8.95	−0.98	7.98	7	280.32	438	17	106.75	87.5	2.5
Jar 6	8.88	−0.89	7.99	7.1	280.32	438	17.1	103.7	85	3.5

**Fig. 1:**
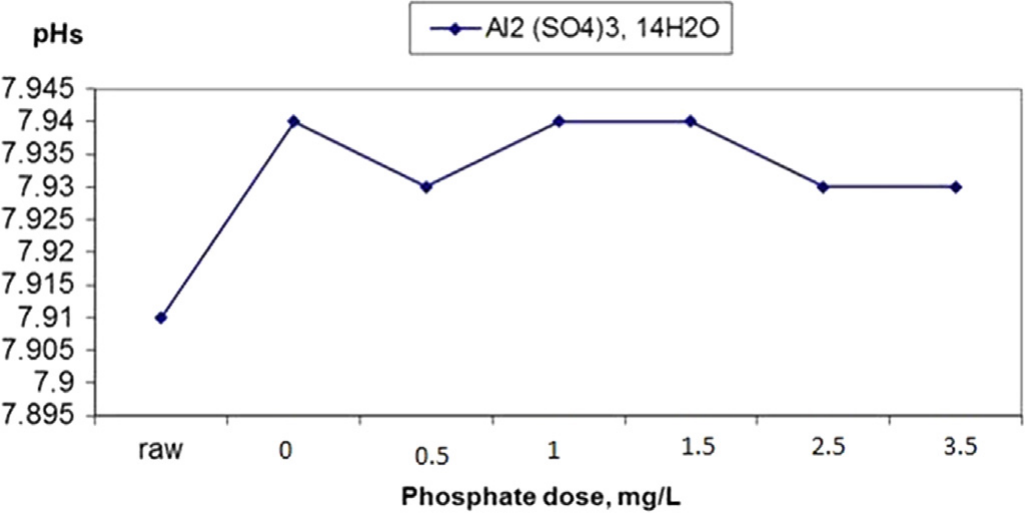
The changes in water pHs in response to increased phosphate dose elevation in coagulation by alum.

**Fig. 2:**
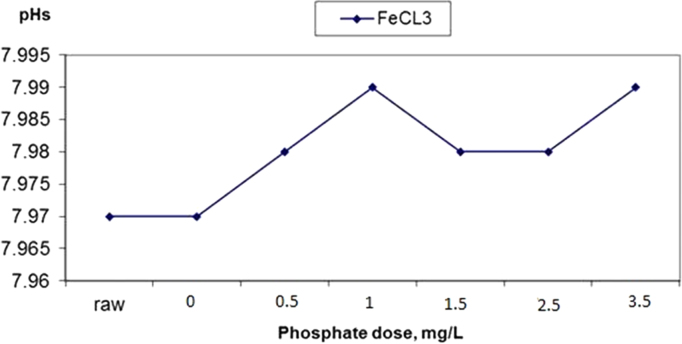
The changes in water pHs in response to increased phosphate dose elevation in coagulation by ferric chloride.

**Fig. 3:**
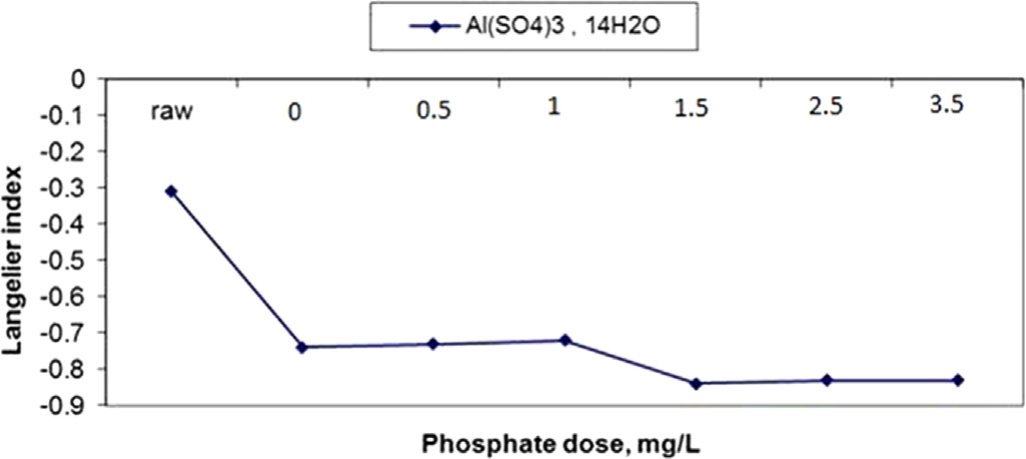
The changes in Langelier saturation index in response to phosphate dose elevation in coagulation by alum.

**Fig. 4:**
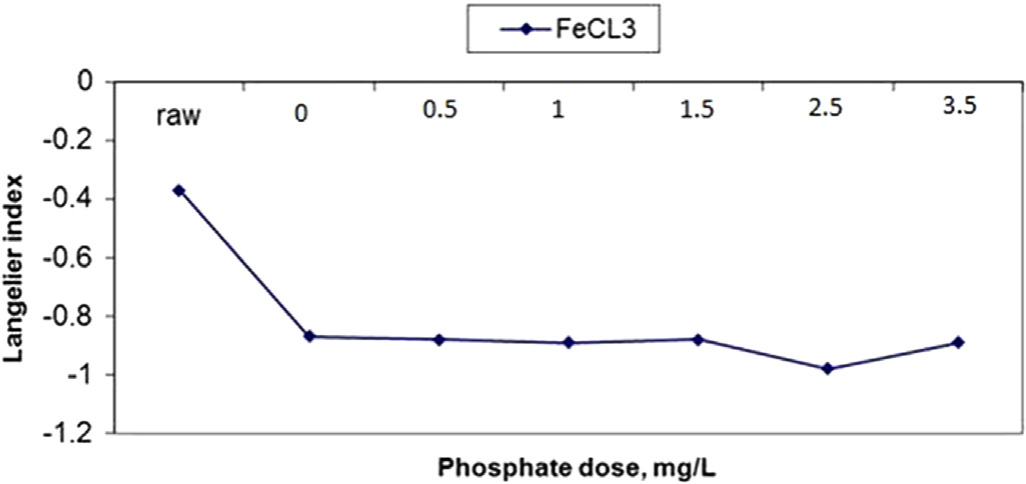
The changes in Langelier saturation index in response to phosphate dose elevation in coagulation by ferric chloride.

**Fig. 5:**
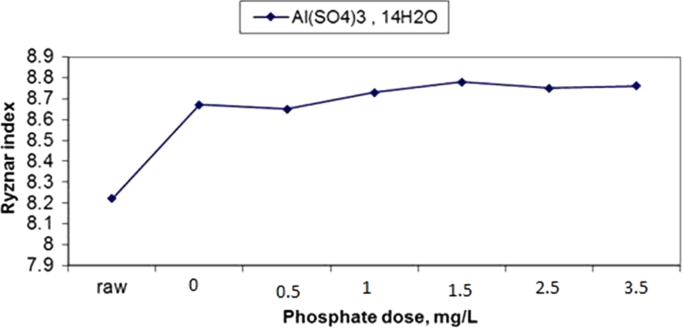
The changes in the Ryznar index in response to phosphate dose elevation in coagulation by alum.

**Fig. 6:**
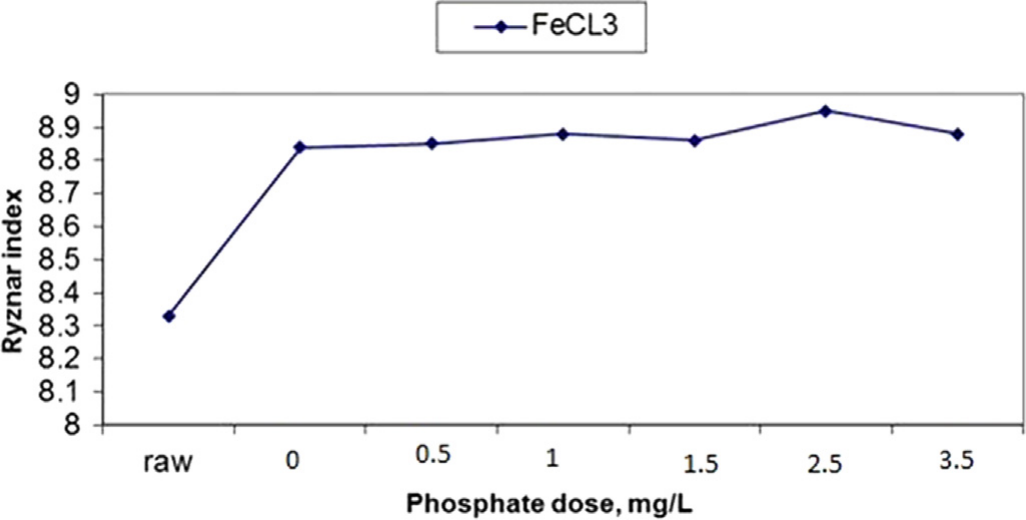
The changes in the Ryznar index in response to phosphate dose elevation in coagulation by ferric chloride.

**Fig. 7:**
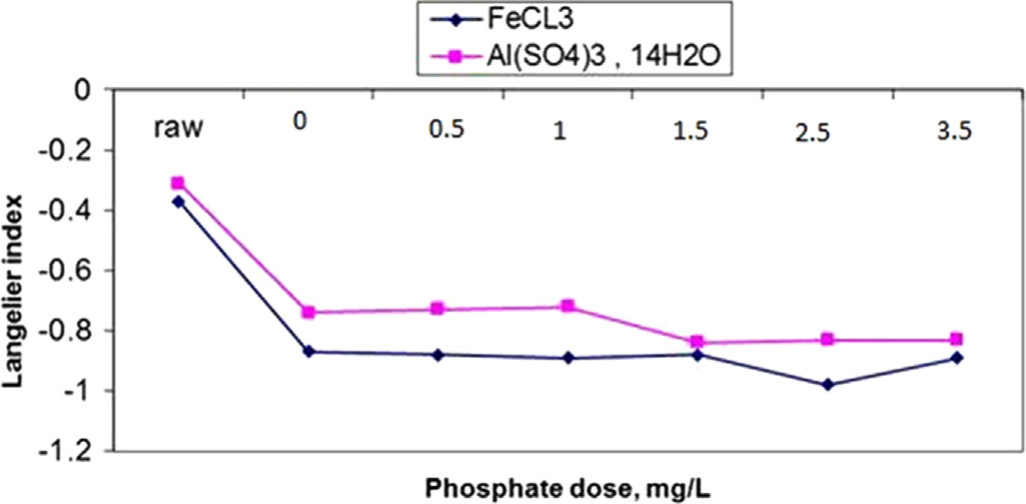
Comparison of changes in Langelier saturation index due to increased phosphate dosage in coagulation with alum and ferric chloride.

**Fig. 8:**
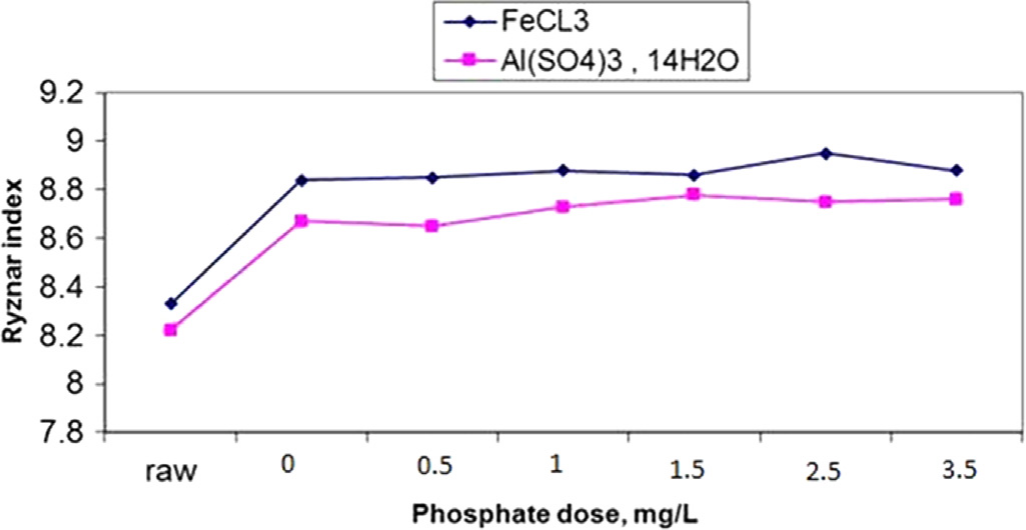
Comparison of changes in Ryznar saturation index due to increased phosphate dosage in coagulation with alum and ferric chloride.

The results of changes in water pHs in response to elevation of phosphate dose in coagulation by alum and ferric chloride are demonstrated in Fig. 1, Fig. 2. The changes in Langelier and Ryznar saturation indices in response to phosphate dose elevation in coagulation by alum and ferric chloride are shown in Fig. 3, Fig. 4, Fig. 5, Fig. 6. The results of comparing the changes in the Langelier and Ryznar saturation indices in response to elevation of phosphate dose in coagulation by alum and ferric chloride are shown in Fig. 7, Fig. 8.

## 2 Experimental design, materials and methods

### 2.1 Chemical materials

All chemicals used, including Na_2_HPO_4_.2H_2_O, 14H_2_O, Al_2_(SO_4_)_3,_ FeCl_3_. 6H_2_O, H_2_SO_4_, CH_3_COOH, and HCl, were purchased from Merck Co., Germany.

### 2.2 Design of experiments

Synthetic water samples were used for comparing the corrosion indices of alum and ferric chloride coagulants in conventional coagulation processes of water with phosphate dose elevation, synthetic water samples were used. To prepare the synthetic water sample, natural clay soil water utilized. For this purpose, first this soil was passed through a sieve with mesh 120 and then 1.5 mg/L of it was weighed by a balance and poured into the water sample. For homogenization, it was then stirred for 10 min. Next, the solution was kept still for 24 h and then stirred again. After 30 min of sedimentation, the top water was used for the experiments.

### 2.3 Jar experiments

The jar experiments (coagulation, flocculation, and sedimentation) were performed by standard jar test device (Sedimentation Jar test, AZTEC Environmental Control LTD) using six 1-L beakers at room temperature. All jars and pedals of mixing were washed by HCl before doing any experiment and then rinsed off by distilled water [13], [14], [15]. Addition of phosphate compounds was also performed during rapid mixing, one minute before or after injecting the coagulant (alum or ferric chloride). The phosphate compounds were injected as 0.5, 1, 1.5, 2, 2.5, 3, and 3.5 mg/L into jar glasses. However, no phosphate was added to any of the jar glasses across all jar tests so that the extent of residual metal in every situation would be determined. Further, 10 mg/L doses of alum and ferric chloride were used for conventional coagulation. Eventually, the jar samples were filtered by a syringe filter with 0.45 μm pores and the phosphate level of the samples was measured by DR/2000 Direct Read Spectrophotometer at the wavelength of 420 nm. Eventually, the Langelier and Ryznar corrosion indices were calculated by the equations in Table 1.

Furthermore, all of the qualitative parameters of the samples including alkalinity, electric conductivity, total suspended solids, temperature, bicarbonate and pH were performed by the instructions in Standard methods for the examination of water and wastewater, 20th edition. The pH of the samples was measured by pH meter (Metrohm Herisau) E520.
